# A role for intestinal TLR4-driven inflammatory response during activity-based anorexia

**DOI:** 10.1038/srep35813

**Published:** 2016-10-25

**Authors:** Liliana Belmonte, Najate Achamrah, Séverine Nobis, Charlène Guérin, Gaëtan Riou, Christine Bôle-Feysot, Olivier Boyer, Vincent Richard, Jean Claude Do Rego, Pierre Déchelotte, Alexis Goichon, Moïse Coëffier

**Affiliations:** 1 Normandie Univ, UR, Rouen, France; 2 INSERM unit 1073, Rouen, France; 3 Institute for Research and Innovation in Biomedicine (IRIB), Rouen, France; 4 Rouen University Hospital, Nutrition Department, Rouen, France; 5 Flow cytometry facility CyFlow, Rouen, France; 6 INSERM, U905, Rouen, France; 7 INSERM, U1096, Rouen, France; 8 Animal Behavior Platform SCAC, Rouen, France

## Abstract

Anorexia nervosa (AN) is associated with low-grade systemic inflammation and altered gut microbiota. However, the molecular origin of the inflammation remains unknown. Toll-like receptors are key regulators of innate immune response and their activation seems also to be involved in the control of food intake. We used activity-based anorexia (ABA) model to investigate the role of TLR4 and its contribution in anorexia-associated low-grade inflammation. Here, we found that ABA affected early the intestinal inflammatory status and the hypothalamic response. Indeed, TLR4 was upregulated both on colonic epithelial cells and intestinal macrophages, leading to elevated downstream mucosal cytokine production. These mucosal changes occurred earlier than hypothalamic changes driving to increased levels of IL-1β and IL-1R1 as well as increased levels of plasma corticosterone. Paradoxically, TLR4-deficient mice exhibited greater vulnerability to ABA with increased mortality rate, suggesting a major contribution of TLR4-mediated responses during ABA-induced weight loss.

Anorexia nervosa (AN) is a serious eating disorder characterized by a significant malnutrition (more than 15% BMI deficit), a fear of gaining weight, and an excessive obsession about body shape and weight^1^. AN is most prevalent in adolescent girls and young women and it is associated with the highest rates of mortality between all psychiatric disorders although more recent studies report less severe mortality rates^2^,^3^. The molecular mechanisms underlying the outset, progression and maintenance of AN remain poorly understood. The concept of microbiota-gut-brain axis in the regulation of food intake has emerged in the last years^4^.

Recent studies have demonstrated the presence of autoantibodies directed against neuropeptides regulating food intake^4^,^5^,^6^,^7^,^8^ and the origin of these autoantibodies seems to be in the gut^9^. In this context, alterations of the gut barrier function could play a key role in the pathophysiology of eating disorders and particularly in AN by increasing endotoxemia and low grade inflammation. We previously reported increased colonic permeability in anorectic male mice^10^. Elevated chemokine and pro-inflammatory cytokine levels have been reported during AN^11^ and plasma levels of TNF-α were correlated to AN duration^12^. Toll-like receptors (TLRs) activation seems also to be involved in the control of food intake^13^. TLRs are key regulators of innate immune response to bacteria and have been proposed to act as a connection between inflammation and metabolism. TLR4 principally recognizes bacterial lipopolysaccharide (LPS) and its activation mainly leads to the synthesis of pro-inflammatory cytokines and chemokines. Studies in animal models demonstrate that mice deficient in TLR4 were protected from high-fat-diet-induced obesity^13^,^14^. It has been shown that TLR4 plays an important role in obesity-associated inflammation and insulin resistance^15^,^16^; however regulation of TLR4 during AN has not been investigated.

Pro-inflammatory cytokines produced both peripherally and centrally can have profound effects on the brain function (e.g, melanocortin system in the hypothalamus) relevant to behavior and possibly can disturb anorexic food comportment^17^,^18^. In addition cytokines can also modulate the release of gastrointestinal hormones that impact food intake regulation^17^. Disturbances of bidirectional gut-brain system have been involved in a wide range of disorders, including obesity and eating disorders. Even if accumulating evidence suggests hypothalamic dysfunction in AN^19^, it is still unclear whether this dysregulation is a primary event or secondary to the starvation and weight loss. Our hypothesis is that alterations of the gut barrier function could activate the intestinal immune response during the early period of food restriction, and could act as a trigger for systemic low-grade inflammation during AN.

Thus, the present study aimed to analyze intestinal TLR4 signaling pathway activation during activity-based anorexia (ABA), as well as the hypothalamic immune response to early weight loss. We used TLR4 knockout (KO) mice to test the effect of TLR4 on ABA induction. We used the ABA model since it is one of the most widely used animal models for the study of AN^20^,^21^.

## Results

### Activity-based anorexia model

#### Physiological and behavioral measures during food restriction

**Figure 1:**
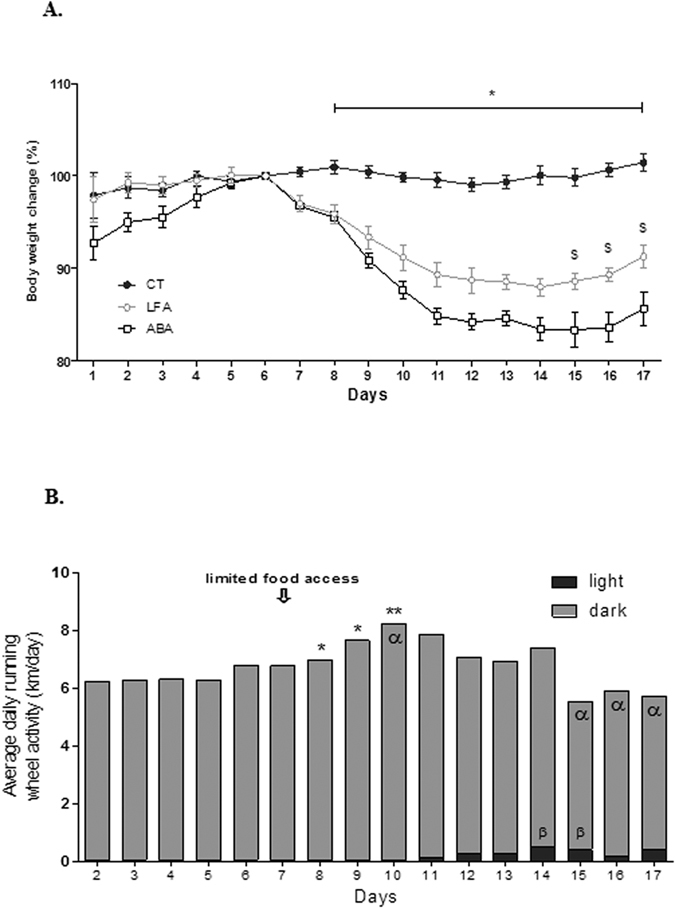
Body weight and wheel activity. (**A**) Percentage of body weight change measured in control mice (CT, closed circles) and in mice placed in standard cages with limitation of food access (LFA, open circles), or in cages with activity wheel and limitation offood access (ABA, open squares). The data represent cumulative body weight loss expressed in %**/**d6. *p < 0.001 vs ABA and LFA; ^$^p < 0.05 vs ABA. (**B**) Wheel activity, expressed in km/day, measured in ABA mice by using RunningWheel® software (Intellibio, France). Total activity and activities during the dark (grey) or light (black) phases were continuously monitored. *p < 0.05 vs d1; **p < 0.01 vs d1; ^α^p < 0.05 vs d7, ^β^p < 0.05 vs d7.

We observed that body weights of female mice remained similar in the three groups (control group *“ad libitum”* (CT), ABA group and control group *“limited food access”* (LFA) during the adaptation phase (d1 to d5, Fig. 1A). However, over the first 5 days of limited food access (d6 to d11), body weight rapidly decreased in ABA and LFA mice. At d11, ABA mice lost 15.2% of their weight compared to d6, whereas LFA mice lost 10.7%. Thereafter, body weight slightly decreased in ABA and LFA mice until d17. We found that weight loss was more marked in ABA compared with LFA mice from d15 to d17 (p < 0.05). Cumulative food intake was similarly reduced in ABA and LFA groups compared with CT whereas cumulative water intake was not affected (data not shown).

We also analyzed the physical activity in ABA mice throughout the adaptation phase (d2 to d6) and we observed that it was mainly present during the dark phase (Fig. 1B). Then, from d7 to d10 (beginning of limited food access), the wheel activity was increased (7.75 km/d at d7 vs 8.45 km/d at d10, p < 0.05). Subsequently from d10, there was a clear trend of decrease in wheel activity but it remained elevated until d17 (%/d6), despite body weight loss. By contrast, activity during the light phase significantly increased at d11 (0.022 km/d at d7 vs 0.14 km/d at d11, p < 0.05) and reached the highest value at d14 (0.4 km/d, p < 0.001 vs d6) (Fig. 1B). Nevertheless, the proportion of physical activity during the light phase remains low related to total activity (7.19%).

#### Paracellular colonic permeability during initial (d10) and persistent weight loss (d17)

**Figure 2:**
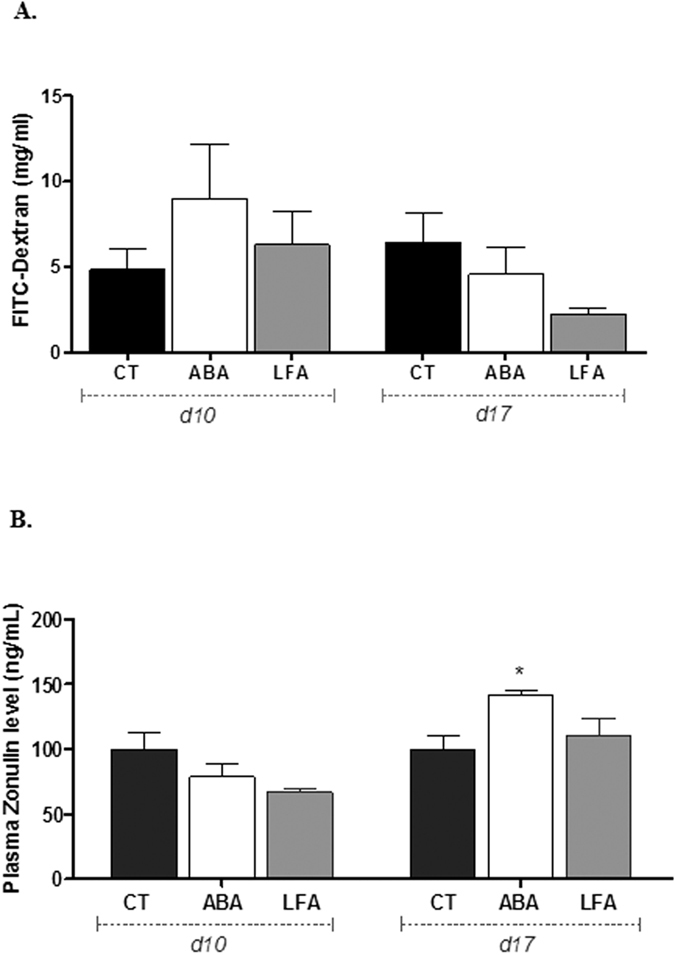
Intestinal Permeability. (**A**) Paracellular permeability measured by FITC-dextran (4 kDa) fluxes assessed in colonic samples from control mice (CT) or mice placed in standard cages with limitation of food access (LFA) or in cages with activity wheel and limitation of food access (ABA) at day 10 and at day 17. (**B**) Plasma zonulin levels were assessed in sera and expressed as ng/ml. *p < 0.05 vs CT.

To determine if gut barrier function is disturbed during the initial period of experimental anorexia, we have evaluated the colonic paracellular permeability by measuring 4 kDa FITC-labeled dextran flux in Ussing chambers. We did not find any differences in the FITC-dextran flux between CT, ABA and LFA groups, neither at d10 nor at d17 (p > 0.05) (Fig. 2A). To further explore, we also evaluated circulating plasma zonulin, the only known physiologic modulator of intercellular tight junctions recently reported as a plasma marker of gut barrier^22^. We observed that during initial weight loss period, levels of circulating zonulin were similar in CT, ABA and LFA groups. However, during persistent weight loss, ABA mice have significant higher levels of plasma zonulin compared with LFA and CT (p < 0.05; Fig. 2B).

#### Differential TLR4 expression during initial and persistent weight loss

**Figure 3:**
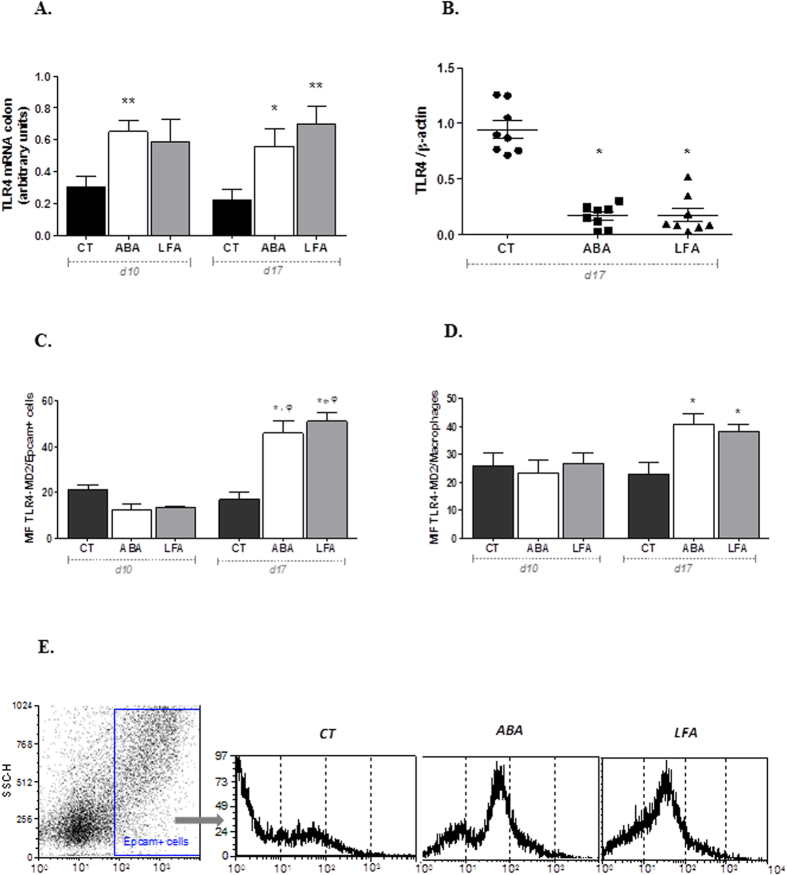
TLR4 expression. (**A**) TLR4 mRNA levels in colonic mucosa of CT, ABA and LFA mice at d10 and d17. Data are shown as mean ± SEM. Results were normalized to the values in GAPDH control amplifications. *p < 0.05 vs CT; **p =  < 0.005 vs CT. (**B**) TLR4 expression protein assessed in colonic samples from CT, ABA and LFA mice.*p < 0.0001 vs CT. (**C**) TLR4–MD-2 expression on epithelial cells (EpCam^+^ cells) isolated from colonic mucosa. MFI, mean fluorescence intensity. *p < 0.001 vs CT; ^φ^p < 0.05 vs d10. (**D**) TLR4–MD-2 expression on macrophages isolated from colonic mucosa. *p < 0.05 vs CT. (**E**) Representative flow cytometry analysis of colonic epithelial cells (EpCam^+^) cells and representative histograms for TLR4 of the outlined population for CT, ABA and LFA.

As TLRs are of critical importance in maintaining gut barrier function and homeostasis, we evaluate colonic TLR4 expression during initial and persistent weight loss (d10 and d17 respectively). We first investigated the gene expression of TLR4 in the whole colonic mucosa of mice. We observed that TLR4 mRNA expression was significantly increased in ABA mice when compared to CT during initial weight loss (d10) and also in ABA and LFA during persistent weight loss (d17) (p < 0.05, Fig. 3A). We next analyzed TLR4 total protein level and we did not find any differences between the 3 groups throughout initial weight loss (d10, data not shown). However, surprisingly, we observed significant lower levels of total TLR4 protein content in ABA and in LFA mice when compared to CT mice during persistent weight loss (d17) (p < 0.05, Fig. 3B). In order to clarify data discrepancies, we assessed TLR4-MD2-complex cell surface expression by flow cytometric analysis in epithelial cells and macrophages from the colonic mucosa. MD-2 is a secreted molecule necessary for TLR4-mediated recognition of LPS. We observed that surface TLR4-MD2 expression on colonic epithelial cells was similar in ABA, LFA and CT mice at d10 (Fig. 3C). Similarly, there were no differences in macrophages-TLR4-MD2 expression between the groups at d10 (Fig. 3D). However, consistent with our previous results, we observed that TLR4-MD2 surface expression on epithelial cells was significantly higher in ABA and LFA mice compared to CT at d17 (p < 0.001 and p = 0.0007, respectively, Fig. 3C). We observed similar results when we analyzed TLR4 expression on CD11b+ F4/80+ cells (macrophages) as TLR4-MD2 expression was increased in ABA and LFA at d17 (p = 0.02 and p = 0.04, respectively, Fig. 3D).

**Figure 4:**
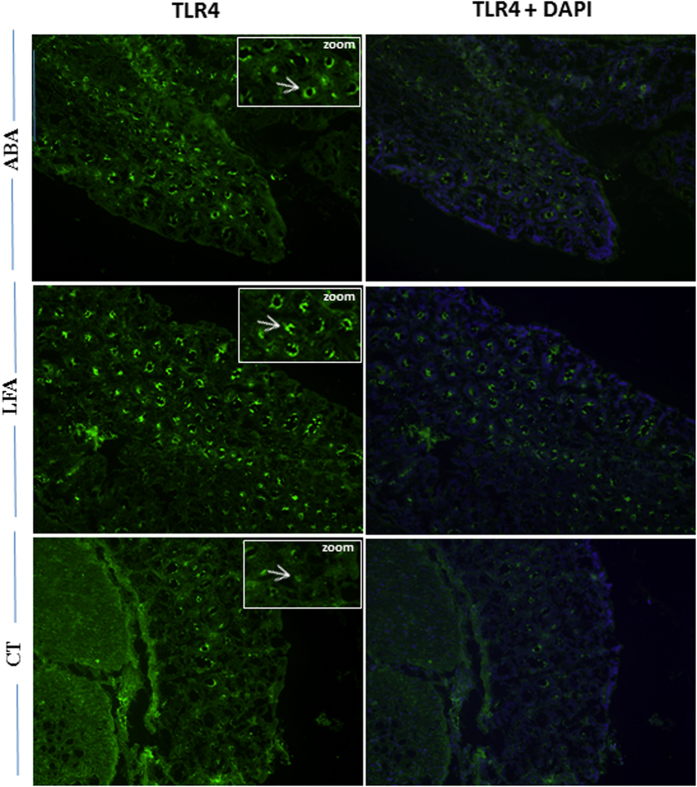
TLR4 expression by immunofluorescence. Representative photomicrographs show the distribution of TLR4 protein in the colonic mucosa of ABA, LFA and CT. Green, TLR4 staining; blue, DAPI nuclear staining. Original magnification, ×20.

Finally, TLR4 expression was explored by immunofluorescence staining in the colon. Most TLR4 expression was localized in the crypts. TLR4 immunofluorescence intensity was markedly enhanced in ABA and LFA when compared to CT during persistent weight loss (d17, Fig. 4).

#### Increased mucosal cytokines expression during ABA

**Figure 5:**
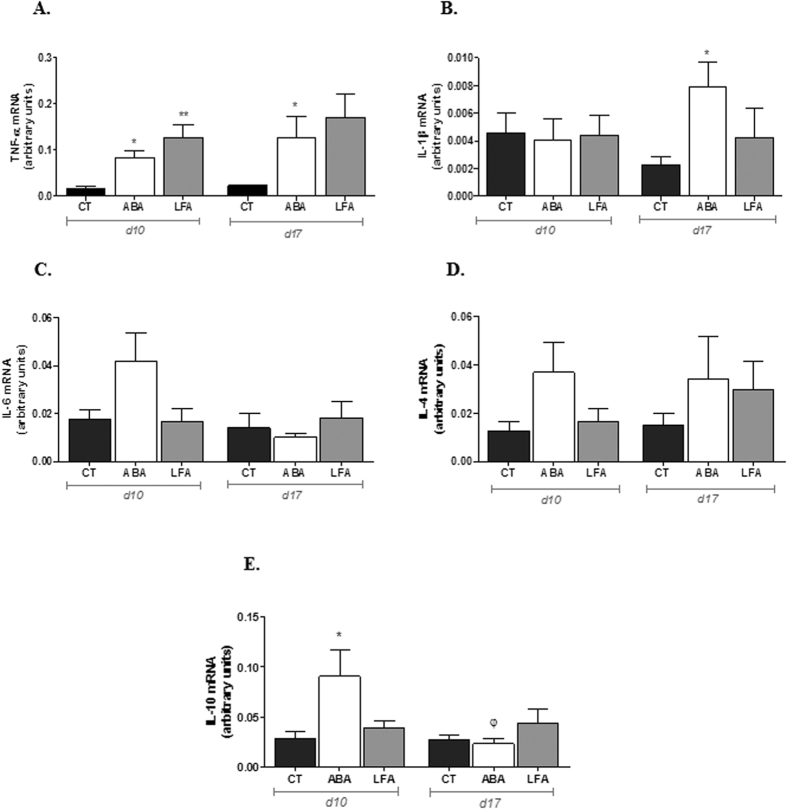
Mucosal cytokine gene expression. (**A**) TNF-α mRNA, (**B**) IL1-β mRNA, (**C**) IL-6 mRNA, (**D**) IL-4 mRNA and (**E**) IL-10 mRNA expression in the colon of CT, ABA and LFA mice at d10 and d17. Data are shown as Mean ± SEM. *p < 0.05 vs CT, **p < 0.01 vs CT, ^φ^p < 0.05 vs d10.

We next asked whether the increased colonic TLR4 expression, was associated to an augmented mucosal innate immune response. Therefore, we examined whole colonic mucosa homogenate from ABA, LFA and CT for cytokine expression both at d10 and at d17 (Fig. 5). Our results revealed significantly increased TNF-α mRNA levels in ABA and LFA compared to CT during initial weight loss and persisted elevated in ABA at d17 (Fig. 5A). IL-1β levels were increased only in the ABA group during persistent weight loss (Fig. 5B) while IL-6 and IL-4 were not significantly affected during both periods (Fig. 5C,D). The expression of the anti-inflammatory cytokine IL-10 was also significantly increased in ABA during initial weight loss (d10) but was restored at d17 (Fig. 5E).

To investigate the downstream signaling pathways in TLR4-mediated inflammation following ABA, we next examined the TIR adaptors expression in the colonic mucosa. We observed that the TIR adaptors of the MyD88-independent pathway, TLR adaptor molecule 1 (TRIF) and TRIF-related adaptor molecule (TRAM) expression were not different between ABA and CT mice (Supplementary Figure S1A,B). However, nuclear NF-κB expression, a downstream event of the TLR4/MyD88 signaling pathway^23^, was significantly increased in the colonic mucosa in response to ABA (Supplementary Figure S1C).

#### TLR4 and the adaptor proteins MyD88, TRIF and TRAM remained unchanged in the hypothalamus during initial and persistent weight loss

**Figure 6:**
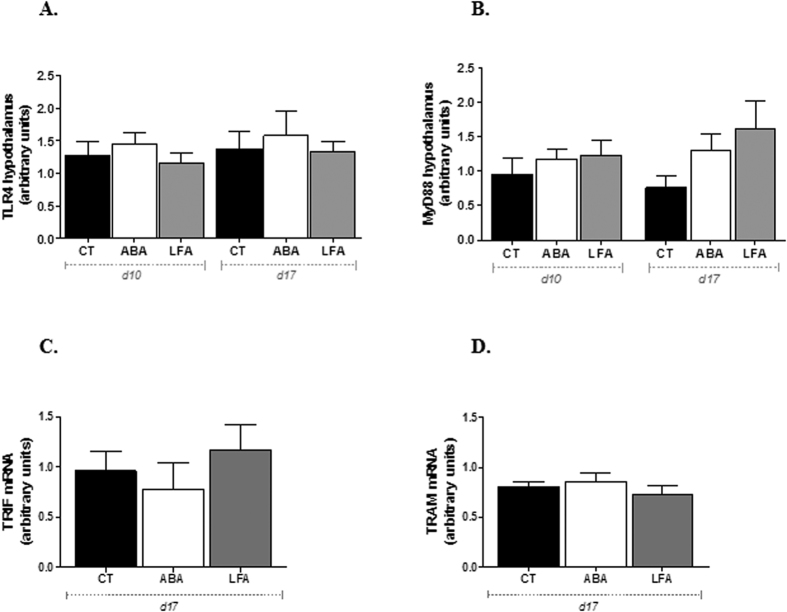
Hypothalamic TLR4, MyD88, TRIF and TRAM expression. (**A**) TLR4 protein expression and (**B**) MyD88 protein expression in the hypothalamus of CT, ABA and LFA mice at d10 and d17, (**C**) TRIF mRNA expression and (**D**) TRAM mRNA expression in the hypothalamus of CT, ABA and LFA mice at d17.

As central immune dysregulation have been described during AN, we hypothesized that hypothalamic TLR4 signaling pathway may possibly be activated in ABA. We assessed TLR4 expression in hypothalamus of ABA, LFA and CT mice and we found that food restriction combined or not with physical activity did not affect TLR4 expression neither at d10 nor at d17 (Fig. 6A). Similar results were obtained for MyD88, a critical adaptor protein in TLR4 signaling pathway, since no significant differences were observed during initial and persistent weight loss, even if a trend towards an increase of MyD88 was observed in ABA and LFA mice at d17 (Fig. 6B). We also assessed the TIR adaptors of the MyD88-independent pathway in the hypothalamus at d17 and we observed that TRIF and TRAM mRNA expressions were not affected in LFA and ABA mice (Fig. 6C,D).

#### Increased hypothalamic expression of IL-1β, IL-1R1 and IRAK-4 during persistent weight loss

**Figure 7:**
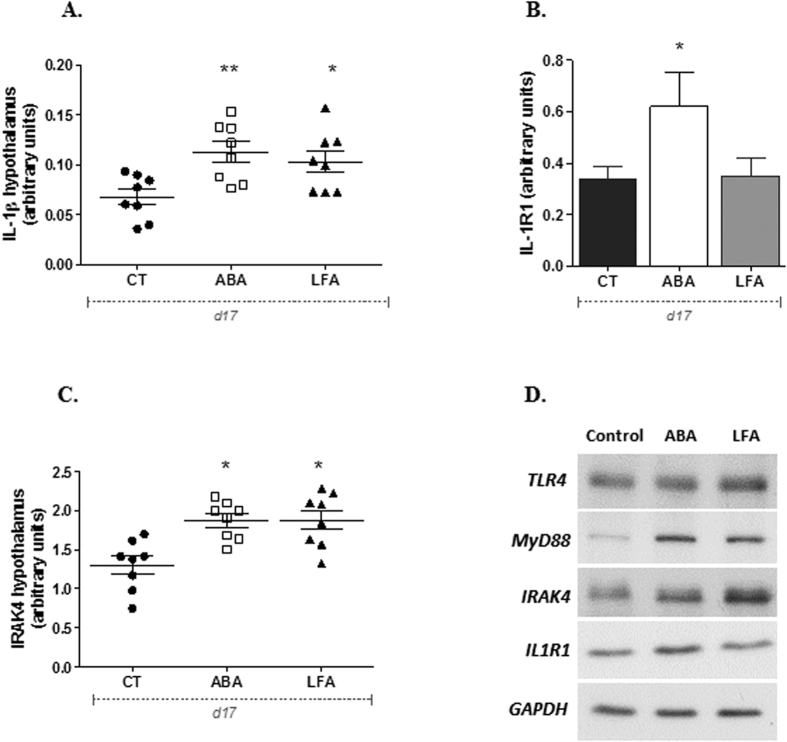
Hypothalamic IL1-β IL-1R1 and IRAK-4 levels. (**A**) IL-1β protein expression, (**D**) IL-1R1 protein expression and (**E**) IRAK-4 protein expression in the hypothalamus of CT, ABA and LFA mice at d17. *p < 0.05 vs CT, **p < 0.01 vs CT. (**F**) Representative immunoblots for TLR4, MyD88, IRAK-4, IL1-R1 and GAPDH from CT, ABA and LFA mice at d17.

Although TLR4 seems not to be activated in the hypothalamus in ABA mice, anyway we aimed to investigate the brain levels of the pro-inflammatory cytokine IL-1β. As IL-1β initiates transduction by binding to IL-1R1, we assessed both proteins in hypothalamic homogenates by western blot. No major changes were observed in the expression of IL-1β and IL-1R1 at d10 (data not shown). However, levels of IL-1β were significantly increased in ABA and LFA compared with CT at d17 (p = 0.004 and p = 0.01, respectively) (Fig. 7A). Likewise, hypothalamic IL-1R1 expression was significantly higher in ABA compared to CT and LFA at d17 (p < 0.05) (Fig. 7B). As the NLRP3 inflammasome activation is a key signaling event for activation and secretion of IL-1β, we also assessed, in the hypothalamus, NLRP3 mRNA levels that were not different between the 3 groups (CT: 0.68 ± 0.1, ABA: 0.68 ± 0.1 and LFA: 0.59 ± 0.07, arbitrary units, p = ns).

To go forward in the signaling cascade, we investigated Interleukin-1 receptor-associated kinase (IRAK-4) that is a key mediator in IL-1β and TLR4 signaling. We did not find any difference in the hypothalamic expression of IRAK-4 at d10, while higher levels of IRAK-4 were observed at d17 in ABA and LFA compared to CT (p = 0.001 and p = 0.004, respectively) (Fig. 7C).

#### Differential PPAR-γ levels in colon but not in hypothalamus during persistent weight loss

**Figure 8:**
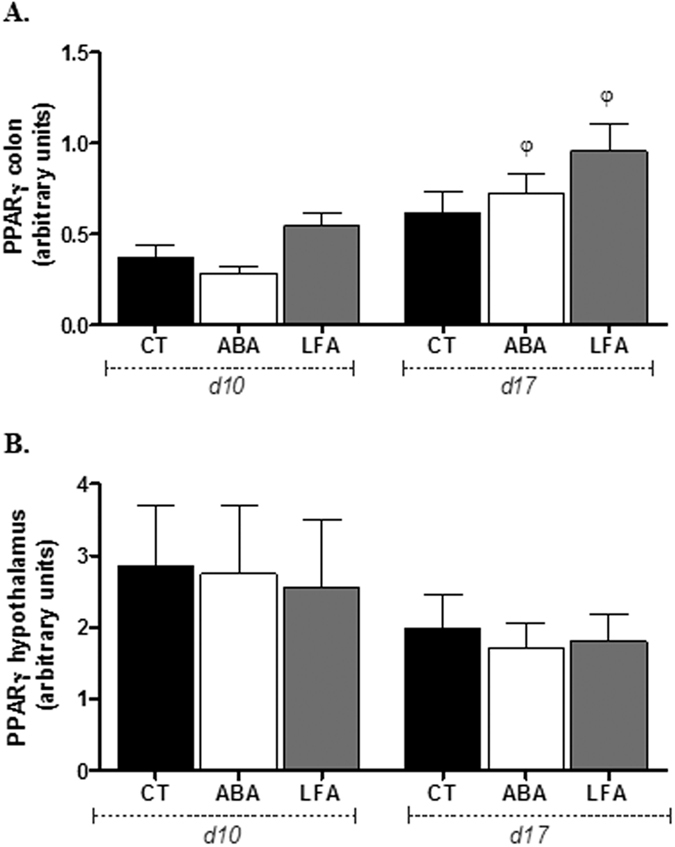
Colonic and hypothalamic PPAR-γ protein expression. (**A**) Colonic PPAR-**γ** expression and (**B**) Hypothalamic PPAR-**γ** expression in CT, ABA and LFA mice at d10 and d17. ^φ^p < 0.05 vs d10.

Peroxisome proliferator-activated receptor-gamma (PPAR-γ) plays a significant role as a modulator of the TLR4-dependent inflammatory pathway, so we wanted to test PPAR-γ expression during both periods of experimental anorexia (d10 and d17) in both compartments, in colon and hypothalamus. We found that colonic PPAR-γ expression was not different between the 3 groups at d10 and at d17. However, PPAR-γ expression increased significantly from d10 to d17 in ABA and LFA (p < 0.05) while in CT, PPAR-γ remained unchanged (Fig. 8A). We also investigated hypothalamic PPAR-γ expression levels and no significant changes were observed between the groups neither during initial nor during persistent weight loss (Fig. 8B).

#### Increased plasma corticosterone levels in ABA

**Figure 9:**
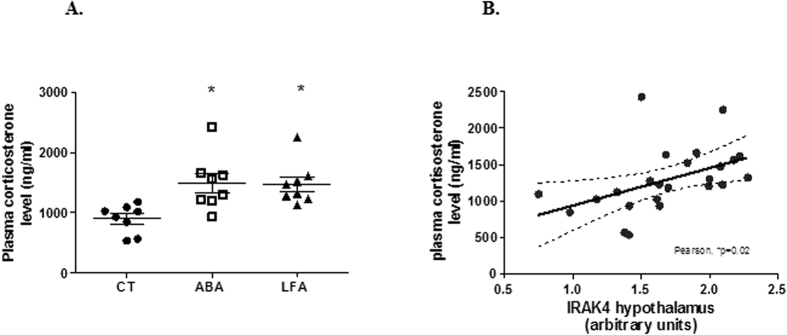
Plasma corticosterone level. (**A**) Plasma corticosterone level in CT, ABA and LFA mice at d17. *p < 0.05 vs CT. (**B**) Correlation between plasma corticosterone and hypothalamic IRAK-4 levels.

Finally, to investigate the impact of weight loss on hypothalamic pituitary axis (HPA) functioning, we assessed plasma corticosterone levels. We observed that plasma corticosterone concentration was elevated in ABA and LFA mice compared to CT at d17 (p = 0.005 and p = 0.001, respectively, Fig. 9A). No changes were observed at d10 (data not shown). Moreover, a significant positive correlation was observed between plasma corticosterone concentration and hypothalamic IRAK-4 levels (p = 0.02, Fig. 9B), suggesting a link between HPA hyperactivity and IL-1β/IL-1R1 signaling activation.

#### Physiological and behavioral measures during ABA induction in ABA-TLR4^−/−^ mice

**Figure 10:**
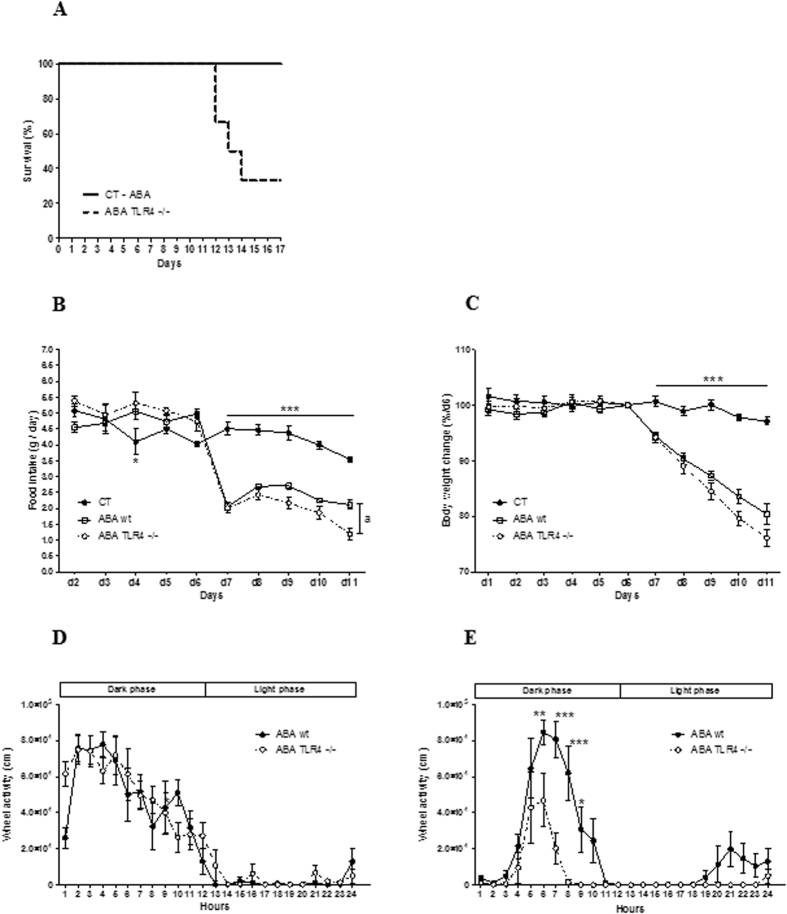
Food intake, body weight and wheel activity of TLR4-KO mice. (**A**) Survival curves for CT (n = 6), ABA wt (n = 6) and ABA TLR4^−/−^ (n = 6) C57BL/6 female mice, *log-rank test, p = 0.0057. (**B**) Food intake and (**C**) Percentage of body weight change measured in control mice (CT, closed circles) and in ABA wild-type (ABA wt, open squares) and ABA TLR4^−/−^, open circles) mice placed in cages with activity wheel and limitation of food access. (**B**) ***p < 0.001 vs ABA wt and ABA TLR4^−/−^, a, p < 0.05. (**C**) The data represents cumulative body weight loss expressed in %**/**d6. ***p < 0.0001 vs ABA wt and ABA TLR4^−/−^. Wheel activity measured at d5 (**D**) and at d11 (**E**), expressed in cm, measured in ABA wt (closed circles) and ABA TLR4^−/−^ (open circles) mice by using RunningWheel® software (Intellibio, France). Activities during the dark or light phases were continuously monitored. *p < 0.05; **p < 0.01; ***p < 0.001. Two-way ANOVA and Bonferroni post-test were used for B, C, D and E.

Finally, to investigate the effects of TLR4 deficiency in the progression of activity-based anorexia, we used wild type (WT) and TLR4^−/−^ mice for ABA induction. Unexpectedly, we observed a high rate of mortality in TLR4^−/−^ mice during ABA compared to WT mice. Indeed, at d12, 4 out of 6 mice were died (Fig. 10A). For this reason, we only reported data on food intake, body weight changes and physical activity until d11.

During the adaptation phase (d1 to d5), food intake and body weight remained similar in CT, ABA WT and ABA TLR4^−/−^ groups. From d6 to d10, food intake decreased significantly in ABA WT and ABA TLR4^−/−^ groups when compared to CT. At d11, food intake was lower in ABA TLR4^−/−^ than in ABA WT (Fig. 10B). Body weight decreased significantly from d7 in ABA WT and ABA TLR4^−/−^ (Fig. 10C). Before the beginning of limited food access (d5), wheel activity was not different between ABA wt and ABA TLR4^−/−^ groups (Fig. 10D). However, at d11, dark phase wheel activity was significantly lower in ABA TLR4^−/−^ mice compared to ABA WT mice (Fig. 10E).

## Discussion

The main finding of the present study is that TLR4 has a dual effect during ABA playing a key role in the early colonic inflammatory response and having a protective role. Our study also report increased hypothalamic IL1-β and IL-1R-1 expression during ABA, which play a central role in the activation of HPA axis acting as essential mediators of the immune-induced glucocorticoid release^24^.

Firstly, we provide evidence for increased TLR4/MD2 expression in colonic epithelial cells and macrophages, and downstream mucosal cytokine production in ABA mice from the beginning of food restriction. Recent studies support the concept that high fat diet and bacteria interact to promote early inflammatory changes in the intestine that contribute to development of or susceptibility to obesity and insulin resistance^16^,^25^. However the potential role of TLR4-mediated response leading to low-grade inflammation during anorexia has received relatively little attention. Although total protein TLR4 levels were reduced in ABA and LFA mice at d17, we could confirm increased TLR4 membrane expression in colonic macrophages and epithelial cells, associated with increased pro-inflammatory cytokines. Activation of TLR4/NF-κB pathway has been previously associated with induced intestinal hyperpermeability^26^. To evaluate intestinal permeability in the present study, we assessed plasma zonulin that is increased in ABA mice at d17, whereas no modifications were observed at the earlier phase (d10). We were not able to confirm these results by evaluating FITC-dextran flux across colonic mucosa in Ussing chambers, while we previously reported increased colonic paracellular permeability in anorectic male mice^10^,^27^. Zonulin has been recently proposed as a marker of intestinal permeability in other diseases like celiac disease, type I diabetes or obesity-associated insulin resistance^28^,^29^. We speculate that zonulin upregulation may precede the onset of gut barrier alteration that may take place later in female mice compared to male mice. Gender-related differences in intestinal permeability after smoking and indomethacin have already been reported by others, showing reduced permeability in women than in men^30^. Further investigations should be done to evaluate whether intestinal permeability is differently affected by sex during ABA. Our results show for the first time TLR4-mediated intestinal inflammatory response associated to the ABA model that reproduces the AN symptoms of body weight loss and physical hyperactivity^20^,^21^. Altered gut microbiota has been previously reported both in AN patients^31^,^32^ and in ABA rats^33^. Associated to a disruption of intestinal barrier, changes in gut microbiota may lead to increased leakage of lipopolysaccharides, which can act upon TLR4 to activate intestinal and systemic inflammation. In a recent meta-analysis, a significant association between anorexia nervosa and increased plasma inflammatory cytokines IL-1β, IL-6 and TNF-α has been reported^34^. Our results are in accordance with the literature as we show increased TNF-α and IL-1β levels in colonic mucosa in ABA mice. TNF-α level augmented earlier than IL-1β. Probably, the anticipated function of TNF-α, induces the others pro-inflammatory cytokines as they act in a cascade pattern, including in the central nervous system^35^,^36^. Furthermore, ABA induction also resulted in increased levels of IL-1β in the hypothalamus suggesting not only intestinal but also hypothalamic inflammation. Peripheral and central pro-inflammatory cytokines lead to satiety through regulation of hypothalamic neuropeptides. In AN, IL-6, IL-1β and TNF-α have anorexigenic effects by regulating peripheral hormones, i.e. leptin, or central mediators like POMC or NPY^37^,^38^. In cancer-induced cachexia models, cytokines signaling is also involved in feeding behavior^39^. Our data suggest that hypothalamic inflammatory response may occur in AN patients.

Secondly, we also found that TLR4 deficiency aggravates the disease since TLR4^−/−^ mice exhibited high mortality rates, suggesting that TLR4 has protective role during ABA. TLR4 activation contributes to the regulation of intestinal inflammatory response but also to the limitation of bacterial translocation by regulating antimicrobial defense, for instance^40^. Indeed, even if TLR4 activation leads to higher intestinal permeability^26^, TLR4 is involved in the regulation of luminal exosome release leading to antimicrobial peptide release^40^. TLR4 deficiency may be thus associated with an altered antimicrobial defense while previous studies reported gut dysbiosis both in AN patients^31^,^32^ and in ABA rats^33^. By contrast, in animal models of obesity, an increased TLR4 expression is shown^16^,^41^ but TLR4 ^−/−^ mice were protected from high-fat diet-induced obesity^13^,^14^.

Our observations significantly add to the emerging role of intestinal TLRs in eating disorders, suggesting that the TLR4 pathway may thus have a dual role, both pathogenic and protective. However, in the present study, we did not evaluate soluble TLR4 that inhibits LPS-induced TLR4 signaling pathway by enabling LPS binding^42^. Soluble TLRs were increased in infectious and non-infectious inflammatory patients^43^, suggesting a key role of soluble TLRs during inflammatory states. Candia *et al*. reported that soluble TLR2 were up-regulated in the colon of patients with ulcerative colitis^44^ but, to our knowledge, soluble TLRs have not yet been more described in intestinal mucosa. Further studies should be performed to evaluate soluble TLRs during anorexia and weight loss.

Activity based anorexia (ABA) is one of the most widely used animal models for the study of AN^20^,^21^,^45^,^46^. This model reproduces hyperactivity behaviors observed in AN patients, as well as reduced food intake, in the presence of hunger, weight loss, desire for activity along with physiological responses of malnutrition^46^. In addition to excessive activity and reduced food intake, weight loss causes the cessation of the estrous cycle in female ABA rats^47^. The ABA model reproduces other clinical manifestations present in AN such as the generalized endocrine disorder that affects the hypothalamus-pituitary-adrenal/gonadal axis^19^,^48^. ABA model may be less appropriate to assess psychopathological disorders even if evaluation of anxiety and/or stress has been previously performed^45^,^49^ Our results should be then confirmed in AN patients.

In conclusion, we show that activity-based anorexia leads to gut inflammation from the initial period of body weight loss through TLR4 activation. Enhanced pro-inflammatory cytokines expression in the gut may be involved in the hypothalamic IL-1β/IL-1R1 signaling pathway activation. However, TLR4 also has protective role since its deficiency leads to high mortality rate. These data underlie the role of gut brain axis during anorexia-induced cachexia that needs to be further investigated.

## Material and Methods

### Animal experimentation and ABA induction

Female C57BL/6 mice used in the study were obtained from Janvier Labs (Le Genest St Isle, France) and acclimatized one week in individual cages at 23 °C with a reversed 12-hour light-dark cycle before study (dark phase: 10:30 AM–10:30 PM). At day 1, 48 female C57BL/6 mice were randomly assigned to 3 experimental groups: ABA group (ABA, *n* = 16), control group named *“limited food access”* (LFA, *n* = 16) and control group *“ad libitum”* (CT, *n* = 16). ABA mice were placed in individual cages with an activity wheel with RunningWheel^®^ software (Intellibio, Seichamps, France). Wheel activity was continuously recorded. LFA and CT mice were placed in individual cages without activity wheel. During 5 days (d1 to d5), all mice had free access to food and water. Then, food access was progressively limited in ABA and LFA groups from 6 h/d at d6 to 3 h/d at d9 until the end of experiment, as previously described^10^. Food was given at the beginning of the dark phase (10:30 AM). Water remained in free access. Body weight, water and food consumption were daily measured at 10:00 AM. In the ABA and LFA groups, water and food intake were also monitored when food was removed. If weight loss exceeded 20% during 3 consecutive days, mice were euthanized for ethical reasons. Two experiments were carried out to evaluate the effects at initial weight loss at d10 and persistent weight loss at d17.

A supplementary experiment was performed with 10-wk-old C57BL/6 female mice knockout for TLR4 (TLR4^−/−^) kindly provided by Dr V Richard, INSERM unit 1096, Rouen University, France. Wild type (wt) controls from the same C57BL/6 background were used for comparisons. Mice were randomly assigned to 3 experimental groups: ABA wt (n = 6), ABA TLR4^−/−^ (n = 6) and Control wt *“ad libitum”* (CT) (n = 6). Behavioral parameters were measured as described before and animals were euthanized at d17. All experiments were carried out in accordance with protocols approved by the local institutional review boards. Animal care and experimentation were carried out in strict accordance with both French and European Community regulations (Official Journal of the European Community L 358, December 18, 1986), M.C. was authorized by the French government to use animal models (authorization no. 76–107). All protocols were approved by the local ethical committee named CENOMEXA (Authorization N/05-11-12/28/11-15).

### Euthanasia and tissue sampling collection

Mice were deeply anesthetized with ketamine/Largactil (40 and 1 mg.kg^−1^, respectively). Blood samples were obtained by puncture in portal vein and collected in 5 ml polyethylene heparinized tubes that were stored on ice until centrifugation. Samples were then centrifuged at 1,500 g for 20 min at 4 °C, and plasma was stored at −80 °C until analysis.

Colon was collected and immediately rinsed with ice-cold PBS. Colon samples were (i) immediately frozen in liquid nitrogen and stored at −80 °C for western blot analysis and quantitative PCR, (ii) embedded in Tissue-Tek O.C.T. compound (Sakura Finetek USA Inc, Torrance, CA), and immediately frozen at −80 °C for immunofluorescence analysis, (iii) freshly processed for flow cytometry and colonic permeability.

Hypothalamus was removed, immediately frozen in liquid nitrogen and stored at −80 °C until analysis.

### Measurement of colonic paracellular permeability

Distal colon samples were removed and cut along the mesenteric border. Colonic permeability was assessed by measuring FITC-dextran (4 kDa) fluxes in Ussing chambers with an exchange surface of 0.07 cm^2^ (Harvard Apparatus, Holliston, MA). FITC-dextran (5 mg/ml) was placed in the mucosal side. After 3 h at 37 °C, medium from the serosal side was removed and stored at −80 °C. The fluorescence level of FITC-dextran (excitation at 485 nm, emission at 535 nm) was measured in 96-well black plate with spectrometer Chameleon V (Hidex Co, Turku, Finland). Values were converted to concentration (mg/mL) using a standard curve.

### Evaluation of serum zonulin

For quantitative assessment of the plasma levels of zonulin, a commercial enzyme-linked immunosorbent assay, Zonulin ELISA Kit, (MyBiosource, San Diego, CA) was used according to the manufacturer’s instructions.

### Total RNA Extraction and real-time qPCR analysis

Total RNA from colon segments was extracted using TRIzol reagent (Invitrogen, Carlsbad, CA). RNA was purified according to the manufacturer’s instructions. Total RNA was treated with DNase I (Invitrogen, Carlsbad, CA) to remove any contaminating DNA. DNase I was removed with DNase inactivation reagent (Invitrogen, Carlsbad, CA) according to the manufacturer’s instructions. The quality and quantity of total RNA were determined using a NanoDrop 2000 spectrophotometer (Thermo Scientific, Wilmington, MA). The ratio of absorbance at 260 nm and 280 nm was used to assess the purity of RNA. A ratio of ≥2.0 was accepted for analysis.

After reverse transcription of 1.5 μg total RNA into cDNA by using 200 units of SuperScript II Reverse Transcriptase (Invitrogen, Carlsbad, CA), quantitative polymerase chain reaction (PCR) was performed by SYBR Green technology on Bio-Rad CFX96 real time PCR system (Bio-Rad Laboratories, Marnes la Coquette, France). GAPDH was chosen as reference gene after checking that similar results were obtained by using three reference genes (GAPDH, β2-microglobulin and 18S RNA, Supplementary Figure S2). All samples were performed in duplicate in a single 96-well reaction plate. Serially diluted cDNA samples were used as external standards. Absolute quantification of mRNA was performed by converting the sample cycle threshold (Ct) values to concentration (copies per ul) based on the standard curves. The identity and purity of the amplified product were assessed by melting curve analysis at the end of amplification. The primer sequences for the targeted mouse genes are presented in Table 1.

### Flow cytometrical analysis

Colon sections were dissected and cut longitudinally after removing the fat and the Peyer’s patches. Small pieces of colon were incubated with EDTA (0.5 M) and dithiothreitol (500 mM) during 20 min at 37 °C with shaking and then processed by mechanical disruption using scissors and passage over a mesh, using previously applied protocols^50^. The resulting mucosal suspension was passed through 70-μm cell strainer and evaluated by flow cytometry. Cells (5 × 10^5^) were incubated with 5 μl/ml purified rat anti-mouse CD16-CD32 mAb (TruStain fcX, Biolegend Inc, San Diego, CA) before incubation with specific anti-mouse antibodies. The following directly conjugated anti-mouse antibodies were used: CD45 PerCP-Cy5.5 (mouse IgG2a, 104 clone), F4/80 APC (rat IgG2a, BM8.1 clone) (both from TONBO, San Diego, CA), CD11b FITC (rat IgG2b, M1/70 clone, Biolegend Inc, San Diego, CA), EpCAM APC (Epithelial cell adhesion molecule) (rat IgG2a, G8.8 clone, LSBio, Seattle, WA) and TLR4/MD-2 complex PE (mouse IgG1, UT15 clone, MBL, Nagoya, Japan). The antibodies were added for 30 min at 4 °C at 2 mg/ml. Cells were washed with PBS and fixed with 0.5% paraformaldehyde. Cells were gated, based on forward and side scatter and on living cells with a FACS Canto (BD Biosciences, San Jose, CA).

Post-acquisition analysis was performed using FCS Express software (DeNovo software, Los Angeles, CA). Acquisition of multiparameter data was carried out with an appropriate forward scatter (FSC) threshold to exclude debris. At least 30,000 intestinal epithelial cells and peritoneal macrophages per sample were analyzed. CD45^+^ cells that were F4/80^+^ CD11b^+^ were considered as macrophages; CD45^−^ EpCam^+^ cells were considered as epithelial cells. Fluorescence intensity was assessed.

### Immunofluorescence

Frozen tissue sections (5 μm thick) were performed using a Leica CM1950 cryostat (Leica Biosystems Nussloch GmbH, Nussloch, Germany) and were mounted on glass slides, and air dried. Tissue was permeabilized for 30 min with PBS solution, 0.2% triton X-100, 1% bovine serum albumin (BSA). After three washes in PBS, nonspecific binding was blocked for 60 min at room temperature with PBS containing 3% (BSA). The sections were then incubated at 4 °C overnight in the same solution supplemented with primary antibodies mouse anti-TLR4 (1:50, Novus biologicals, Littleton, CO). After three washes with PBS, sections were incubated with FITC coupled secondary antibodies (Life Technologies, Cergy-Pontoise, France) for 1 h at room temperature. Negative controls were assessed omitting the primary antibodies. Slides were mounted with Vectashield Mounting Medium 4′,6′-diamidino-2-phenylindole (DAPI) solution (Vector Laboratories, Cambridgeshire, UK) to visualize cell nuclei. After immunohistochemistry, microphotographs were acquired with an Axiolmager Z1 microscope by using Axiovisio software (Carl Zeiss, Gottingen, Germany). Staining for comparative studies was performed in the same experimental session.

### Protein extraction and western blot analysis

For total protein extraction, colon and hypothalamus sections were homogenized at 4 °C in lysis buffer (100 μl Buffer A X2, 2 μl dithiothreitol 100 mM, 50 μl NP40 1%, 1 μl protease inhibitors P8340 1X, 2μl phosphatase inhibitors P2850 1X, for H_2_O 200 μl) (≈100 mg tissue/200 μl lysis buffer).Vials were placed on ice for 15 min and then centrifuged at 12,000 g for 15 min at 4 °C. The supernatant containing proteins was collected and stored at −80 °C until analysis. Proteins (25 μg) were separated on a 4–20% gradient polyacrylamide gel (Bio-Rad, Marnes la Coquette, France) and then transferred to a nitro-cellulose membrane (GE Healthcare, Orsay, France), which was blocked for 1 h at room temperature with 5% (w/v) non-fat dry milk in Tris buffered saline/0.05% Tween 20 (TBS-T) (blocking solution). Then, an overnight incubation at 4 °C was done with mouse anti- TLR4 (working dilution = 1:1000; Novus biologicals, Littleton, CO), PPAR-γ (working dilution = 1:1,000; Fisher Scientific, Illkirch, France), IL-1β, MyD88, NfκB p65 and IRAK-4 (working dilution = 1:1000, Santa Cruz Biotechnology, Tebu-Bio, Le Perray en Yvelines, France). All antibodies were diluted in blocking solution. After three washes in blocking solution, 1 h incubation with peroxidase conjugated goat anti-rabbit or anti-mouse IgG (working dilution = 1:5,000; Santa Cruz Biotechnology, Tebu-Bio, Le Perray en Yvelines, France) was performed. After three additional washes, immunocomplexes were revealed by using the ECL detection system (GE Healthcare Life Sciences, Little Chalfont, UK). Protein bands were quantified by densitometry using ImageScanner III and ImageQuant TL software (GE Healthcare Life Sciences, Little Chalfont, UK).

### Corticosterone assay

A commercial serum corticosterone enzyme immunoassay kit (Abnova, Ann Arbor, MI) was used. All procedures were performed according to the manufacturer’s instructions. Final values were determined by averaging the results of duplicate.

### Statistical analysis

Data are expressed as mean ± standard error mean. Differences between two groups were assessed using the unpaired two-tailed Student’s t-test or non-parametric Mann–Whitney test, as appropriate, and ANOVA followed by Tukey post-test or Kruskall Wallis followed by Dunn’s multiple comparison tests for more than two groups. Pearson analysis was used to analyze correlations between the groups. Survival rates curves were created by Kaplan-Meier method and compared by log-Rank test. Data were analysed using GraphPad Prism version 5.0 for windows (GraphPad software, San Diego, CA). Results were considered statistically significant when p < 0.05.

## Additional Information

**How to cite this article**: Belmonte, L. *et al*. A role for intestinal TLR4-driven inflammatory response during activity-based anorexia. *Sci. Rep.* **6**, 35813; doi: 10.1038/srep35813 (2016).

## Supplementary Material

Supplementary Information

## Figures and Tables

**Table 1:** Sequences of primers used for PCR.

Gene	Sequences	Annealing Temperature (°C)
TLR4	Sense: 5′-AGATCTGAGCTTCAACCCCTTG-3′ Antisense: 5′-AGAGGTGGTGTAAGCCATGC-3′	60
TNF-α	Sense: 5′-TGTCTACTCCTCAGAGCCCC-3′ Antisense 5‘-TGAGTCCTTGATGGTGGTGC-3′	60
IL-1β	Sense: 5′-CCCAAAAGATGAAGGGCTGC-3′ Antisense 5′-AAGGTCCACGGGAAAGACAC-3′	59
IL-6	Sense: 5′-CACTTCACAAGTCGGAGGCT-3′ Antisense 5′-CTGCAAGTGCATCATCGTTGT-3′	59
IL-4	Sense: 5′-ATGGATGTGCCAAACGTCCT-3′ Antisense 5′-TGCAGCTCCATGAGAACACT-3′	59
IL-10	Sense: 5′-ACCTGGTAGAAGTGATGCCC-3′ Antisense 5′-GCTCCACTGCCTTGCTCTTAT-3′	59
NLRP3	Sense: 5′-ACCAGCCAGAGTGGAATGAC-3′ Antisense: 5′-ATGGAGATGCGGGAGAGATA-3′	61
TRIF	Sense: 5′-CAGAGTTGTCTACAAAGTCG-3′ Antisense: 5′-TGGATGACGTGGTGTTCTGC-3′	60
TRAM	Sense: 5′-ATAAAGCTCCCTCGTCTGCC-3′ Antisense: 5′-GGTGTGTGCTCGGTTTCAGG-3′	63
GAPDH	Sense: 5′-ATCACTGCCACTCAGAAGA-3′ Antisense 5′-AAGTCACAGGAGACAACCT-3′	57
