# Supplementary material for: A role for intestinal TLR4-driven inflammatory response during activity-based anorexia
Source: Sci Rep. 2016 Oct 25;6:35813. doi: 10.1038/srep35813 (PMC5078809; doi:10.1038/srep35813)
Supplement: Supplementary Information [file srep35813-s1.doc]

**A role for intestinal TLR4-driven inflammatory response during activity-based anorexia.**

Liliana Belmonte^1,2,3,4*^, Najate Achamrah^1,2,3,4*^, Séverine Nobis^1,2,3^, Charlène Guérin^1,2,3^, Gaëtan Riou^1,3,5^, Christine Bôle-Feysot^1,2,3^, Olivier Boyer^1,3,5,6^, Vincent Richard^1,3,7^, Jean Claude Do Rego^1,3,8^, Pierre Déchelotte^1,2,3,4^, Alexis Goichon^1,2,3^, Moïse Coëffier^1,2,3,4^.

^1^ Normandie Univ, UR, Rouen, France.

^2^ INSERM unit 1073, Rouen, France

^3^ Institute for Research and Innovation in Biomedicine (IRIB), Rouen, France

^4^ Rouen University Hospital, Nutrition Department, Rouen, France

^5^ Flow cytometry facility CyFlow, Rouen, France.

^6^ INSERM, U905, Rouen, France

^7^ INSERM, U1096, Rouen, France

^8^ Animal Behavior Platform SCAC, Rouen, France

**Supplementary Figure S1**

**TRIF, TRAM and NF-κB expression in the colon.**

**A.**

**B.**

**C.**

(A) TRIF mRNA expression, and (B) TRAM mRNA expression in the colon of CT, ABA and LFA mice at d17. (C) NF-κB expression protein assessed in colonic samples from CT, ABA and LFA mice at d17.*p<0.01 vs CT.

**Supplementary Figure S2**

**TLR4 mRNA levels in the colonic mucosa at day 17 normalized by a single reference gene and by multiple references genes.**

A

B

(A) TLR4/GAPDH ratio (p<0.05, ANOVA) and (B) TLR4/mean of 3 references genes ratio (p<0.05, ANOVA). The 3 reference genes used are GAPDH, β2-microglobulin and 18S RNA.
